# (1*RS*,6*SR*)-Ethyl 4-(4-chloro­phen­yl)-6-(4-fluoro­phen­yl)-2-oxocyclo­hex-3-ene-1-carboxyl­ate toluene hemisolvate

**DOI:** 10.1107/S1600536811000158

**Published:** 2011-01-12

**Authors:** Grzegorz Dutkiewicz, B. Narayana, K. Veena, H. S. Yathirajan, Maciej Kubicki

**Affiliations:** a Department of Chemistry, Adam Mickiewicz University, Grunwaldzka 6, 60-780 Poznań, Poland; b Department of Studies in Chemistry, Mangalore University, Mangalagangotri 574 199, India; c Department of Studies in Chemistry, University of Mysore, Manasagangotri, Mysore 570 006, India

## Abstract

In the crystal structure of the title compound, C_21_H_18_ClFO_3_·0.5C_7_H_8_, the toluene solvent mol­ecules occupy special positions on centres of symmetry, and consequently are disordered across this site. The cyclo­hexene ring has a slightly distorted sofa conformation; the two benzene rings are inclined by 72.90 (7)° and their planes make dihedral angles of 30.09 (10) (chloro­phen­yl) and 88.13 (6)° (fluoro­phen­yl) with the approximately planar part of the cyclo­hexenone ring [maximum deviation from plane through five atoms is 0.030 (2) Å, the sixth atom is 0.672 (3)Å out of this plane]. Weak inter­molecular C—H⋯O and C—H⋯*X* (*X* = F, Cl) inter­actions join mol­ecules into a three-dimensional structure. Also, a relatively short and directional C—Cl⋯F—C contact is observed [Cl⋯F = 3.119 (2) Å, C—Cl⋯F = 157.5 (2)° and C—F⋯Cl 108.3 (2)°]. The solvent mol­ecules fill the voids in the crystal structure and are kept there by relatively short and directional C—H⋯π inter­actions.

## Related literature

For biological applications of some cyclo­hexa­nones, see: Eddington *et al.* (2000 ▶). For asymmetry parameters, see: Duax & Norton (1975 ▶). For similar structures, see: in Anuradha *et al.* (2009 ▶); Fun *et al.* (2008 ▶, 2009 ▶, 2010 ▶); Badshah *et al.* (2009 ▶). For a description of the Cambridge Structural Database, see: Allen (2002 ▶).
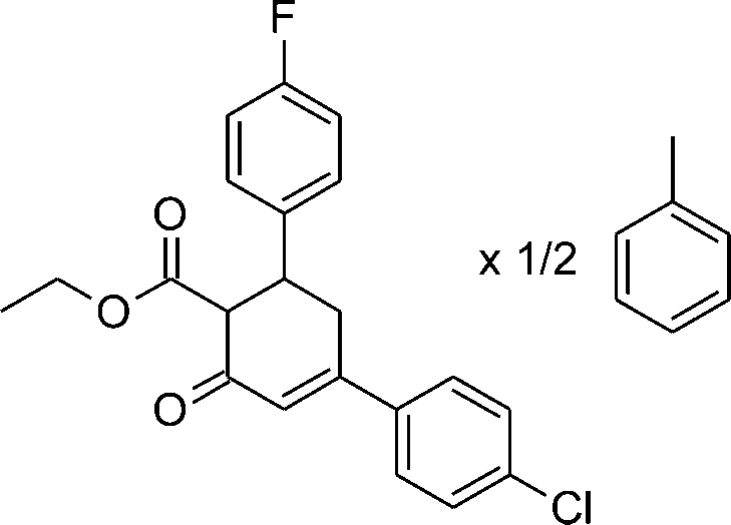


## Experimental

### 

#### Crystal data

C_21_H_18_ClFO_3_·0.5C_7_H_8_
*M* *_r_* = 418.87
Triclinic, 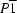

*a* = 7.572 (2) Å
*b* = 11.259 (3) Å
*c* = 13.362 (3) Å
α = 69.42 (2)°
β = 86.58 (2)°
γ = 70.98 (2)°
*V* = 1006.3 (4) Å^3^
*Z* = 2
Mo *K*α radiation
μ = 0.22 mm^−1^
*T* = 100 K
0.3 × 0.25 × 0.1 mm

#### Data collection

Oxford Diffraction Xcalibur Eos diffractometer
Absorption correction: multi-scan (*CrysAlis PRO*; Oxford Diffraction, 2009 ▶) *T*_min_ = 0.990, *T*_max_ = 1.000
8414 measured reflections
4154 independent reflections
2567 reflections with *I* > 2σ(*I*)
*R*_int_ = 0.030

#### Refinement

*R*[*F* ^2^ > 2σ(*F* ^2^)] = 0.042
*wR*(*F* ^2^) = 0.085
*S* = 1.02
4154 reflections
341 parameters
H atoms treated by a mixture of independent and constrained refinement
Δρ_max_ = 0.24 e Å^−3^
Δρ_min_ = −0.28 e Å^−3^

### 

Data collection: *CrysAlis PRO* (Oxford Diffraction, 2009 ▶); cell refinement: *CrysAlis PRO*; data reduction: *CrysAlis PRO*; program(s) used to solve structure: *SIR92* (Altomare *et al.*, 1993 ▶); program(s) used to refine structure: *SHELXL97* (Sheldrick, 2008 ▶); molecular graphics: *SHELXTL* (Sheldrick, 2008 ▶); software used to prepare material for publication: *SHELXL97*.

## Supplementary Material

Crystal structure: contains datablocks I, global. DOI: 10.1107/S1600536811000158/dn2647sup1.cif

Structure factors: contains datablocks I. DOI: 10.1107/S1600536811000158/dn2647Isup2.hkl

Additional supplementary materials:  crystallographic information; 3D view; checkCIF report

## Figures and Tables

**Table 1:** Hydrogen-bond geometry (Å, °) *Cg* is the centroid of the C1*A*–C3*A*,C1*A*′–C3*A*′ ring.

*D*—H⋯*A*	*D*—H	H⋯*A*	*D*⋯*A*	*D*—H⋯*A*
C45—H45⋯F64^i^	0.94 (2)	2.54 (2)	3.327 (3)	141.6 (15)
C5—H52⋯F64^ii^	0.938 (19)	2.54 (2)	3.432 (3)	159.3 (15)
C6—H6⋯Cl44^iii^	1.003 (19)	2.84 (2)	3.846 (3)	176.3 (14)
C65—H65⋯O12^iv^	0.94 (2)	2.59 (2)	3.519 (3)	173.6 (16)
C3—H3⋯*Cg*	0.918 (19)	2.78 (2)	3.627 (3)	155.0 (17)
C3—H3⋯*Cg*^v^	0.918 (19)	2.78 (2)	3.627 (3)	155.0 (17)

Symmetry codes: (i) 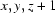; (ii) 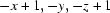; (iii) 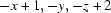; (iv) 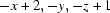; (v) 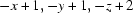.

**Fig. 1.**
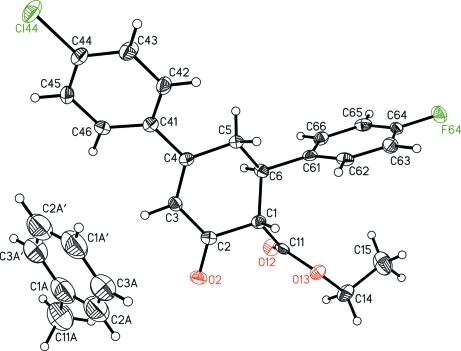
Anisotropic ellipsoid representation of the components of I together with atom labelling scheme. The ellipsoids are drawn at 50% probability level, hydrogen atoms are depicted as spheres with arbitrary radii; only one of the disordered toluene molecules is shown. [Symmetry code: (i) 1 - x,1 - y,2 - z]

**Fig. 2.**
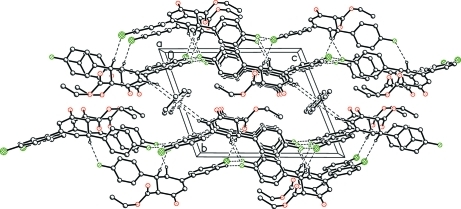
The crystal packing as seen along x-direction. Weak interactions (cf. text) are shown as dashed lines. For the sake of clarity, H atoms not involved in hydrogen interactions have been omitted .

## supplementary crystallographic information

### Comment

Cyclohexenone derivatives, prepared either from natural sources or entirely *via* synthetic routes, are known to possess a wide variety of biological activities, *e.g.* they were reported to have anticonvulsant, antimalarial, anti-inflammatory and cardiovascular effects (Eddington *et al.*, 2000). In the course of our studies on chalcone derivatives, we have synthesized some cyclohexene derivatives. Structures of some similar compounds have been reported earlier (for instance, ethyl 6-(4-chlorophenyl)-4-(4-methoxyphenyl)- 2-oxocyclohex-3-ene-1- carboxylate, Fun *et al.*, 2009, ethyl 4-(4-methoxyphenyl)-2-oxo-6- phenylcyclohex-3-ene-1-carboxylate, Fun *et al.*, 2008, ethyl 4-(4-bromophenyl)-6-(4-ethoxyphenyl)- 2-oxocyclohex-3-enecarboxylate, Badshah *et al.*, 2009). Here we report the crystal structure of (1RS,6SR) ethyl 4-(4-chlorophenyl)-6-(4-fluorophenyl)-2-oxocyclohex-3-ene-1-carboxylate toluene solvate (I, Scheme 1).

The overall conformation of I (Fig. 1) can be characterized by the dihedral angles between the phenyl rings, of 72.90 (7)°, and between these rings and the plane of cyclohexene ring which are equal to 30.09 (10)° for chlorophenyl ring and 88.13 (6)° for fluorophenyl ring. These values are similar to those found in the structures of related compounds, for instance in methyl 4,6-bis(4-fluorophenyl)-2-oxocyclohex- 3-ene-1-carboxylate (Fun *et al.*, 2010) the dihedral angles between fluorophenyl rings in two symmetry-independent molecules are 79.7 (2)° and 73.7 (2)°, and the angles between the cyclohexene plane and the fluorophenyl rings are 14.9° and 73.7° in one molecule and 29.9° and 84.0° in the second. In the structure of ethyl 6 - r-(2-chlorophenyl)-2-oxo-4- phenylcyclohex-3-ene-1 - t-carboxylate (Anuradha *et al.*, 2009) appropriate angles are 81.73 (12)°. 12.75 (14)° and 74.16 (8)°.

The cyclohexene ring adopts slightly distorted sofa conformation, the asymmetry parameter Δ*C_s_*^3^ (Duax & Norton, 1975) is 6.2°. This is also confirmed by least-squares calculations: five atoms C1 - C5 are almost coplanar, maximum deviation is 0.030 (2) Å, while the sixth atom, C6, is by 0.672 (3)Å out of this mean plane.

In the crystal structure the molecules are joined by weak C—H···O, C—H···F and C—H···Cl interactions (Fig. 2). The solvent - toluene molecules are disordered over the centre of symmetry. They occupy the voids in the crystal structure and are kept there by means of relatively short and linear C—H···π interactions (H···*Cg* 2.78 Å, C—H···*Cg* 155°). An inteeresting feature of the structure is the presence of linear C—Cl···F—C contacts (F···Cl 3.12 Å, C—Cl···F 157.5 (2)°, C—F···Cl 108.3 (2)°). In the CSD (Allen, 2002) there are 196 cases of such contacts shorter than 3.2 Å, and the same directional preferences are observed.

### Experimental

A mixture of ((2*E*)-1-(4-chlorophenyl)-3-(4-fluorophenyl)prop-2-en-1-one (0.01 mol) and ethyl acetoacetate (0.01 mol) were refluxed for 2 hr in 10–15 ml of ethanol in the presence of 0.8 ml 10% NaOH. The crystals were obtained by a slow evaporation from toluene solution. C_21_H_18_ClFO_3_.C_7_H_8_: C: 72.26 (72.33); H:5.59 (5.64); m.p. 346 K.

### Refinement

Hydrogen atoms from solvent molecule were located geometrically (*C*(methyl)-H 0.98 Å, C(arom)-H 0.95 Å) and refined as a riding model; the *U*_iso_ values of H atoms were set at 1.2 (1.5 for CH_3_ group) times *U*_eq_ of their carrier atom. All other hydrogen atoms were located in difference Fourier maps and isotropically refined.

### Crystal data

**Table:**

C_21_H_18_ClFO_3_·0.5C_7_H_8_	*Z* = 2
*M**_r_* = 418.87	*F*(000) = 438
Triclinic, *P*1	*D*_x_ = 1.382 Mg m^−^^3^
Hall symbol: -P 1	Mo *K*α radiation, λ = 0.71073 Å
*a* = 7.572 (2) Å	Cell parameters from 4263 reflections
*b* = 11.259 (3) Å	θ = 2.9–28.2°
*c* = 13.362 (3) Å	µ = 0.22 mm^−^^1^
α = 69.42 (2)°	*T* = 100 K
β = 86.58 (2)°	Plate, colourless
γ = 70.98 (2)°	0.3 × 0.25 × 0.1 mm
*V* = 1006.3 (4) Å^3^	

### Data collection

**Table:**

Oxford Diffraction Xcalibur Eos diffractometer	4154 independent reflections
Radiation source: Enhance (Mo) X-ray Source	2567 reflections with *I* > 2σ(*I*)
graphite	*R*_int_ = 0.030
Detector resolution: 16.1544 pixels mm^-1^	θ_max_ = 28.2°, θ_min_ = 2.9°
ω scans	*h* = −9→10
Absorption correction: multi-scan (*CrysAlis PRO*; Oxford Diffraction, 2009)	*k* = −14→14
*T*_min_ = 0.990, *T*_max_ = 1.000	*l* = −17→12
8414 measured reflections	

### Refinement

**Table:**

Refinement on *F*^2^	Primary atom site location: structure-invariant direct methods
Least-squares matrix: full	Secondary atom site location: difference Fourier map
*R*[*F*^2^ > 2σ(*F*^2^)] = 0.042	Hydrogen site location: inferred from neighbouring sites
*wR*(*F*^2^) = 0.085	H atoms treated by a mixture of independent and constrained refinement
*S* = 1.02	*w* = 1/[σ^2^(*F*_o_^2^) + (0.032*P*)^2^] where *P* = (*F*_o_^2^ + 2*F*_c_^2^)/3
4154 reflections	(Δ/σ)_max_ < 0.001
341 parameters	Δρ_max_ = 0.24 e Å^−^^3^
0 restraints	Δρ_min_ = −0.28 e Å^−^^3^

### Special details

**Table:**

Geometry. All e.s.d.'s (except the e.s.d. in the dihedral angle between two l.s. planes) are estimated using the full covariance matrix. The cell e.s.d.'s are taken into account individually in the estimation of e.s.d.'s in distances, angles and torsion angles; correlations between e.s.d.'s in cell parameters are only used when they are defined by crystal symmetry. An approximate (isotropic) treatment of cell e.s.d.'s is used for estimating e.s.d.'s involving l.s. planes.
Refinement. Refinement of *F*^2^ against ALL reflections. The weighted *R*-factor *wR* and goodness of fit *S* are based on *F*^2^, conventional *R*-factors *R* are based on *F*, with *F* set to zero for negative *F*^2^. The threshold expression of *F*^2^ > σ(*F*^2^) is used only for calculating *R*-factors(gt) *etc*. and is not relevant to the choice of reflections for refinement. *R*-factors based on *F*^2^ are statistically about twice as large as those based on *F*, and *R*- factors based on ALL data will be even larger.

### Fractional atomic coordinates and isotropic or equivalent isotropic displacement parameters (Å^2^)

**Table:**

	*x*	*y*	*z*	*U*_iso_*/*U*_eq_	Occ. (<1)
C1	0.6617 (3)	0.3346 (2)	0.63392 (17)	0.0170 (5)	
H1	0.553 (3)	0.4099 (19)	0.5898 (14)	0.014 (5)*	
C11	0.8347 (3)	0.3354 (2)	0.57104 (17)	0.0174 (5)	
O12	0.98805 (19)	0.25572 (14)	0.60237 (11)	0.0245 (4)	
O13	0.79236 (18)	0.43431 (14)	0.47543 (11)	0.0194 (3)	
C14	0.9412 (3)	0.4356 (3)	0.40032 (19)	0.0251 (5)	
H141	0.921 (3)	0.529 (2)	0.3592 (17)	0.032 (6)*	
H142	1.060 (3)	0.398 (2)	0.4422 (15)	0.020 (5)*	
C15	0.9245 (4)	0.3639 (4)	0.3282 (2)	0.0443 (8)	
H151	1.031 (3)	0.364 (2)	0.2731 (19)	0.047 (7)*	
H152	0.939 (3)	0.267 (3)	0.369 (2)	0.052 (9)*	
H153	0.812 (4)	0.395 (3)	0.290 (2)	0.063 (9)*	
C2	0.6800 (3)	0.3601 (2)	0.73669 (16)	0.0173 (5)	
O2	0.77611 (19)	0.42695 (14)	0.74167 (11)	0.0235 (4)	
C3	0.5696 (3)	0.3083 (2)	0.82386 (17)	0.0175 (5)	
H3	0.577 (3)	0.3302 (19)	0.8831 (15)	0.017 (5)*	
C4	0.4587 (3)	0.23905 (19)	0.81810 (15)	0.0155 (5)	
C41	0.3380 (3)	0.19687 (19)	0.90529 (16)	0.0166 (5)	
C42	0.1667 (3)	0.1872 (2)	0.88281 (18)	0.0218 (5)	
H42	0.132 (3)	0.2043 (19)	0.8152 (15)	0.012 (5)*	
C43	0.0493 (3)	0.1525 (2)	0.96266 (17)	0.0255 (6)	
H43	−0.066 (3)	0.150 (2)	0.9474 (17)	0.042 (7)*	
C44	0.1033 (3)	0.1251 (2)	1.06689 (17)	0.0223 (5)	
Cl44	−0.04482 (8)	0.08177 (6)	1.16805 (4)	0.03293 (18)	
C45	0.2740 (3)	0.1303 (2)	1.09298 (18)	0.0203 (5)	
H45	0.311 (3)	0.110 (2)	1.1647 (16)	0.021 (6)*	
C46	0.3900 (3)	0.1664 (2)	1.01258 (17)	0.0187 (5)	
H46	0.511 (3)	0.1664 (19)	1.0331 (14)	0.020 (5)*	
C5	0.4500 (3)	0.2036 (2)	0.71992 (17)	0.0168 (5)	
H51	0.339 (3)	0.2683 (19)	0.6691 (15)	0.017 (5)*	
H52	0.439 (3)	0.118 (2)	0.7395 (15)	0.016 (5)*	
C6	0.6248 (3)	0.2016 (2)	0.65685 (17)	0.0180 (5)	
H6	0.734 (3)	0.1305 (19)	0.7046 (15)	0.017 (5)*	
C61	0.6118 (3)	0.1733 (2)	0.55508 (16)	0.0159 (5)	
C62	0.4812 (3)	0.2627 (2)	0.47154 (17)	0.0192 (5)	
H62	0.397 (3)	0.344 (2)	0.4785 (14)	0.016 (5)*	
C63	0.4697 (3)	0.2382 (2)	0.37815 (18)	0.0214 (5)	
H63	0.381 (3)	0.301 (2)	0.3201 (16)	0.027 (6)*	
C64	0.5913 (3)	0.1206 (2)	0.37091 (16)	0.0191 (5)	
F64	0.57885 (17)	0.09459 (12)	0.27937 (9)	0.0284 (3)	
C65	0.7231 (3)	0.0291 (2)	0.45003 (17)	0.0205 (5)	
H65	0.803 (3)	−0.050 (2)	0.4421 (15)	0.018 (6)*	
C66	0.7323 (3)	0.0571 (2)	0.54264 (17)	0.0184 (5)	
H66	0.821 (3)	−0.0050 (19)	0.5957 (15)	0.011 (5)*	
C1A	0.6922 (4)	0.4700 (3)	1.0220 (2)	0.0570 (8)	
H1A	0.8218	0.4493	1.0377	0.068*	0.50
C11A	0.8853 (4)	0.4547 (3)	1.0355 (2)	0.0601 (17)	0.50
H11A	0.9479	0.4489	0.9703	0.072*	0.50
H11B	0.9449	0.3725	1.0962	0.072*	0.50
H11C	0.8954	0.5322	1.0494	0.072*	0.50
C2A	0.6030 (5)	0.5692 (3)	0.9267 (3)	0.0566 (8)	
H2A	0.6723	0.6166	0.8762	0.068*	
C3A	0.4143 (5)	0.5989 (3)	0.9052 (2)	0.0561 (8)	
H3A	0.3552	0.6673	0.8400	0.067*	

### Atomic displacement parameters (Å^2^)

**Table:**

	*U*^11^	*U*^22^	*U*^33^	*U*^12^	*U*^13^	*U*^23^
C1	0.0164 (11)	0.0172 (12)	0.0182 (12)	−0.0066 (10)	0.0027 (9)	−0.0063 (10)
C11	0.0206 (12)	0.0163 (12)	0.0215 (13)	−0.0096 (11)	0.0015 (10)	−0.0108 (11)
O12	0.0191 (8)	0.0237 (9)	0.0271 (9)	−0.0028 (7)	0.0009 (7)	−0.0084 (7)
O13	0.0156 (8)	0.0206 (8)	0.0203 (8)	−0.0064 (7)	0.0050 (6)	−0.0051 (7)
C14	0.0183 (12)	0.0311 (15)	0.0233 (14)	−0.0107 (12)	0.0081 (10)	−0.0052 (12)
C15	0.0387 (18)	0.075 (3)	0.0380 (18)	−0.0286 (18)	0.0177 (14)	−0.0350 (18)
C2	0.0142 (11)	0.0149 (11)	0.0219 (12)	−0.0038 (10)	−0.0005 (9)	−0.0059 (10)
O2	0.0249 (8)	0.0262 (9)	0.0277 (9)	−0.0157 (8)	0.0064 (7)	−0.0135 (7)
C3	0.0190 (11)	0.0180 (12)	0.0177 (12)	−0.0063 (10)	0.0017 (9)	−0.0085 (10)
C4	0.0143 (11)	0.0127 (11)	0.0176 (12)	−0.0032 (9)	0.0007 (9)	−0.0040 (9)
C41	0.0160 (11)	0.0128 (11)	0.0185 (12)	−0.0023 (10)	0.0020 (9)	−0.0050 (10)
C42	0.0217 (12)	0.0268 (13)	0.0148 (13)	−0.0088 (11)	−0.0009 (10)	−0.0035 (11)
C43	0.0144 (12)	0.0354 (15)	0.0234 (14)	−0.0099 (11)	0.0011 (10)	−0.0048 (12)
C44	0.0220 (12)	0.0228 (13)	0.0207 (13)	−0.0081 (10)	0.0068 (10)	−0.0061 (11)
Cl44	0.0256 (3)	0.0469 (4)	0.0230 (3)	−0.0148 (3)	0.0094 (2)	−0.0070 (3)
C45	0.0239 (12)	0.0205 (13)	0.0146 (13)	−0.0051 (10)	0.0001 (10)	−0.0059 (10)
C46	0.0184 (12)	0.0173 (12)	0.0218 (13)	−0.0068 (10)	−0.0006 (10)	−0.0073 (10)
C5	0.0179 (12)	0.0165 (12)	0.0173 (12)	−0.0081 (10)	0.0019 (9)	−0.0053 (10)
C6	0.0167 (11)	0.0194 (12)	0.0209 (12)	−0.0073 (10)	0.0017 (9)	−0.0095 (10)
C61	0.0149 (11)	0.0176 (12)	0.0187 (12)	−0.0103 (10)	0.0050 (9)	−0.0065 (10)
C62	0.0161 (11)	0.0180 (12)	0.0269 (13)	−0.0075 (10)	0.0051 (9)	−0.0107 (11)
C63	0.0178 (12)	0.0243 (13)	0.0216 (13)	−0.0080 (11)	−0.0005 (10)	−0.0063 (11)
C64	0.0227 (12)	0.0293 (13)	0.0160 (12)	−0.0183 (11)	0.0084 (9)	−0.0128 (10)
F64	0.0349 (8)	0.0383 (8)	0.0244 (7)	−0.0190 (7)	0.0084 (6)	−0.0202 (6)
C65	0.0184 (12)	0.0182 (12)	0.0285 (14)	−0.0083 (11)	0.0100 (10)	−0.0118 (11)
C66	0.0162 (11)	0.0167 (12)	0.0190 (12)	−0.0049 (10)	0.0000 (9)	−0.0028 (10)
C1A	0.061 (2)	0.064 (2)	0.065 (2)	−0.0231 (19)	0.0106 (18)	−0.043 (2)
C11A	0.052 (4)	0.060 (4)	0.071 (4)	−0.007 (3)	−0.001 (3)	−0.035 (4)
C2A	0.065 (2)	0.060 (2)	0.061 (2)	−0.0264 (19)	0.0088 (18)	−0.0355 (19)
C3A	0.073 (2)	0.052 (2)	0.055 (2)	−0.0239 (19)	0.0092 (17)	−0.0289 (17)

### Geometric parameters (Å, °)

**Table:**

C1—C11	1.514 (3)	C46—H46	0.973 (19)
C1—C2	1.520 (3)	C5—C6	1.524 (3)
C1—C6	1.534 (3)	C5—H51	1.01 (2)
C1—H1	0.996 (19)	C5—H52	0.938 (19)
C11—O12	1.202 (2)	C6—C61	1.517 (3)
C11—O13	1.338 (2)	C6—H6	1.003 (19)
O13—C14	1.464 (2)	C61—C66	1.389 (3)
C14—C15	1.491 (3)	C61—C62	1.390 (3)
C14—H141	0.97 (2)	C62—C63	1.383 (3)
C14—H142	0.98 (2)	C62—H62	0.96 (2)
C15—H151	1.06 (2)	C63—C64	1.377 (3)
C15—H152	1.01 (3)	C63—H63	0.96 (2)
C15—H153	0.91 (3)	C64—C65	1.366 (3)
C2—O2	1.225 (2)	C64—F64	1.369 (2)
C2—C3	1.456 (3)	C65—C66	1.391 (3)
C3—C4	1.340 (3)	C65—H65	0.94 (2)
C3—H3	0.918 (19)	C66—H66	0.921 (19)
C4—C41	1.478 (3)	C1A—C2A	1.392 (4)
C4—C5	1.510 (3)	C1A—C3A^i^	1.408 (4)
C41—C42	1.395 (3)	C1A—C11A	1.4305
C41—C46	1.401 (3)	C1A—H1A	0.9500
C42—C43	1.379 (3)	C11A—H11A	0.9800
C42—H42	0.892 (18)	C11A—H11B	0.9800
C43—C44	1.373 (3)	C11A—H11C	0.9800
C43—H43	0.92 (2)	C2A—C3A	1.381 (4)
C44—C45	1.383 (3)	C2A—H2A	0.9500
C44—Cl44	1.742 (2)	C3A—C1A^i^	1.408 (4)
C45—C46	1.380 (3)	C3A—H3A	0.9500
C45—H45	0.94 (2)		
			
C11—C1—C2	111.06 (17)	C41—C46—H46	121.1 (11)
C11—C1—C6	110.02 (17)	C4—C5—C6	112.59 (17)
C2—C1—C6	111.44 (17)	C4—C5—H51	112.3 (11)
C11—C1—H1	108.1 (10)	C6—C5—H51	107.3 (10)
C2—C1—H1	107.0 (10)	C4—C5—H52	110.4 (12)
C6—C1—H1	109.1 (10)	C6—C5—H52	107.0 (11)
O12—C11—O13	124.93 (18)	H51—C5—H52	107.0 (15)
O12—C11—C1	124.19 (19)	C61—C6—C5	112.59 (17)
O13—C11—C1	110.86 (17)	C61—C6—C1	111.90 (17)
C11—O13—C14	116.65 (16)	C5—C6—C1	108.92 (17)
O13—C14—C15	109.77 (18)	C61—C6—H6	109.7 (11)
O13—C14—H141	105.4 (12)	C5—C6—H6	107.8 (10)
C15—C14—H141	110.0 (13)	C1—C6—H6	105.6 (10)
O13—C14—H142	107.7 (11)	C66—C61—C62	118.12 (19)
C15—C14—H142	114.2 (12)	C66—C61—C6	120.58 (19)
H141—C14—H142	109.3 (17)	C62—C61—C6	121.30 (19)
C14—C15—H151	110.8 (12)	C63—C62—C61	121.6 (2)
C14—C15—H152	111.7 (15)	C63—C62—H62	119.0 (11)
H151—C15—H152	106.4 (19)	C61—C62—H62	119.4 (11)
C14—C15—H153	116.4 (17)	C64—C63—C62	117.7 (2)
H151—C15—H153	108 (2)	C64—C63—H63	121.0 (12)
H152—C15—H153	103 (2)	C62—C63—H63	121.4 (12)
O2—C2—C3	123.01 (19)	C65—C64—F64	118.58 (19)
O2—C2—C1	120.32 (18)	C65—C64—C63	123.4 (2)
C3—C2—C1	116.57 (18)	F64—C64—C63	118.0 (2)
C4—C3—C2	123.6 (2)	C64—C65—C66	117.6 (2)
C4—C3—H3	121.6 (12)	C64—C65—H65	120.7 (12)
C2—C3—H3	114.7 (12)	C66—C65—H65	121.6 (12)
C3—C4—C41	122.00 (18)	C61—C66—C65	121.6 (2)
C3—C4—C5	120.58 (18)	C61—C66—H66	121.2 (12)
C41—C4—C5	117.40 (17)	C65—C66—H66	117.2 (12)
C42—C41—C46	117.75 (18)	C2A—C1A—C3A^i^	118.4 (3)
C42—C41—C4	120.68 (18)	C2A—C1A—C11A	114.00 (18)
C46—C41—C4	121.57 (18)	C3A^i^—C1A—C11A	127.52 (19)
C43—C42—C41	121.5 (2)	C2A—C1A—H1A	120.8
C43—C42—H42	119.0 (12)	C3A^i^—C1A—H1A	120.8
C41—C42—H42	119.5 (12)	C1A—C11A—H11A	109.5
C44—C43—C42	119.3 (2)	C1A—C11A—H11B	109.5
C44—C43—H43	119.2 (14)	H11A—C11A—H11B	109.5
C42—C43—H43	121.4 (14)	C1A—C11A—H11C	109.5
C43—C44—C45	121.08 (19)	H11A—C11A—H11C	109.5
C43—C44—Cl44	119.46 (16)	H11B—C11A—H11C	109.5
C45—C44—Cl44	119.46 (17)	C3A—C2A—C1A	120.3 (3)
C46—C45—C44	119.4 (2)	C3A—C2A—H2A	119.9
C46—C45—H45	119.9 (12)	C1A—C2A—H2A	119.9
C44—C45—H45	120.7 (12)	C2A—C3A—C1A^i^	121.3 (3)
C45—C46—C41	121.0 (2)	C2A—C3A—H3A	119.4
C45—C46—H46	117.9 (11)	C1A^i^—C3A—H3A	119.4
			
C2—C1—C11—O12	63.8 (3)	C42—C41—C46—C45	−1.1 (3)
C6—C1—C11—O12	−60.1 (3)	C4—C41—C46—C45	178.40 (19)
C2—C1—C11—O13	−117.79 (19)	C3—C4—C5—C6	23.3 (3)
C6—C1—C11—O13	118.35 (18)	C41—C4—C5—C6	−157.89 (18)
O12—C11—O13—C14	6.5 (3)	C4—C5—C6—C61	−176.97 (18)
C1—C11—O13—C14	−171.86 (17)	C4—C5—C6—C1	−52.3 (2)
C11—O13—C14—C15	94.2 (3)	C11—C1—C6—C61	−54.7 (2)
C11—C1—C2—O2	28.6 (3)	C2—C1—C6—C61	−178.39 (17)
C6—C1—C2—O2	151.70 (18)	C11—C1—C6—C5	−179.88 (17)
C11—C1—C2—C3	−154.95 (18)	C2—C1—C6—C5	56.5 (2)
C6—C1—C2—C3	−31.9 (3)	C5—C6—C61—C66	−115.4 (2)
O2—C2—C3—C4	177.6 (2)	C1—C6—C61—C66	121.5 (2)
C1—C2—C3—C4	1.3 (3)	C5—C6—C61—C62	65.2 (2)
C2—C3—C4—C41	−175.33 (19)	C1—C6—C61—C62	−57.9 (2)
C2—C3—C4—C5	3.4 (3)	C66—C61—C62—C63	0.0 (3)
C3—C4—C41—C42	148.1 (2)	C6—C61—C62—C63	179.37 (18)
C5—C4—C41—C42	−30.6 (3)	C61—C62—C63—C64	0.8 (3)
C3—C4—C41—C46	−31.4 (3)	C62—C63—C64—C65	−1.1 (3)
C5—C4—C41—C46	149.9 (2)	C62—C63—C64—F64	179.03 (16)
C46—C41—C42—C43	1.8 (3)	F64—C64—C65—C66	−179.54 (16)
C4—C41—C42—C43	−177.7 (2)	C63—C64—C65—C66	0.5 (3)
C41—C42—C43—C44	−0.9 (3)	C62—C61—C66—C65	−0.5 (3)
C42—C43—C44—C45	−0.7 (3)	C6—C61—C66—C65	−179.92 (18)
C42—C43—C44—Cl44	179.81 (17)	C64—C65—C66—C61	0.3 (3)
C43—C44—C45—C46	1.3 (3)	C3A^i^—C1A—C2A—C3A	0.4 (4)
Cl44—C44—C45—C46	−179.14 (16)	C11A—C1A—C2A—C3A	−177.18 (19)
C44—C45—C46—C41	−0.4 (3)	C1A—C2A—C3A—C1A^i^	−0.4 (4)

Symmetry codes: (i) −*x*+1, −*y*+1, −*z*+2.

### Hydrogen-bond geometry (Å, °)

**Table:**

Cg is the centroid of the C1A–C3A,C1A'–C3A' ring.

**Table:**

*D*—H···*A*	*D*—H	H···*A*	*D*···*A*	*D*—H···*A*
C45—H45···F64^ii^	0.94 (2)	2.54 (2)	3.327 (3)	141.6 (15)
C5—H52···F64^iii^	0.938 (19)	2.54 (2)	3.432 (3)	159.3 (15)
C6—H6···Cl44^iv^	1.003 (19)	2.84 (2)	3.846 (3)	176.3 (14)
C65—H65···O12^v^	0.94 (2)	2.59 (2)	3.519 (3)	173.6 (16)
C3—H3···Cg	0.918 (19)	2.78 (2)	3.627 (3)	155.0 (17)
C3—H3···Cg^i^	0.918 (19)	2.78 (2)	3.627 (3)	155.0 (17)

Symmetry codes: (ii) *x*, *y*, *z*+1; (iii) −*x*+1, −*y*, −*z*+1; (iv) −*x*+1, −*y*, −*z*+2; (v) −*x*+2, −*y*, −*z*+1; (i) −*x*+1, −*y*+1, −*z*+2.
